# Does emotional intelligence predict breaking bad news skills in pediatric interns? A pilot study

**DOI:** 10.3402/meo.v20.24245

**Published:** 2015-08-17

**Authors:** Suzanne Reed, Karyn Kassis, Rollin Nagel, Nicole Verbeck, John D. Mahan, Richard Shell

**Affiliations:** 1 Department of Pediatrics, The Ohio State University College of Medicine, Nationwide Children's Hospital, Columbus, OH, USA; 2 Office of Evaluation, Curricular Research and Development, The Ohio State University College of Medicine, Columbus, OH, USA

**Keywords:** breaking bad news, emotional intelligence, pediatrics

## Abstract

**Background:**

While both patients and physicians identify communication of bad news as an area of great challenge, the factors underlying this often complex task remain largely unknown. Emotional intelligence (EI) has been positively correlated with good general communication skills and successful leadership, but there is no literature relating EI to the delivery of bad news.

**Purpose:**

Our objectives were to determine: 1) performance of first-year pediatric residents in the delivery of bad news in a standardized patient (SP) setting; and 2) the role of EI in these assessments. Our hypothesis was that pediatric trainees with higher EI would demonstrate more advanced skills in this communication task.

**Methods:**

Forty first- year residents participated. Skill in bad news delivery was assessed via SP encounters using a previously published assessment tool (GRIEV_ING Death Notification Protocol). Residents completed the Emotional and Social Competency Inventory (ESCI) as a measure of EI.

**Results:**

Residents scored poorly on bad news delivery skills but scored well on EI. Intraclass correlation coefficients indicated moderate to substantial inter-rater reliability among raters using the delivering bad news assessment tool. However, no correlation was found between bad news delivery performance and EI.

**Conclusions:**

We concluded that first-year pediatric residents have inadequate skills in the delivery of bad news. In addition, our data suggest that higher EI alone is not sufficient to effectively deliver death news and more robust skill training is necessary for residents to gain competence and acquire mastery in this important communication domain.

The ability to communicate bad news is an essential part of medical practice, and a failure to perform this task competently can dramatically and permanently affect the doctor–patient relationship (1–3). Patients and physicians alike identify the communication of bad news as an area of challenge (4–7), and delivering bad news is a source of significant stress for even experienced clinicians (8, 9). In a 2009 study of novice and experienced physicians, delivering bad news was shown to be stressful even in a simulated encounter. Inexperience was correlated with a higher stress response, and poor performance was correlated with burnout and fatigue (8).

Although the importance of good communication is generally understood, many residency and fellowship training programs lack formal instruction on the delivery of bad news, leaving this critical element of training largely to chance. In surveys of residents, fellows, and program directors, the need and desire for further training in communication skills, specifically breaking bad news, is well documented (10–12). The teaching of communication skills in undergraduate medical training varies greatly by program (13, 14), and trainees enter residency programs with mixed skills and experience. Moreover, since many are unlikely to have had significant hands-on experience in delivering bad news, residency training often provides the first real clinical exposure to this challenging task.

Emotional intelligence (EI) is commonly understood as one's ability to recognize own emotions and the emotions of others, and to use this understanding to successfully navigate important interactions (15). EI can influence many facets of an individual's life, both personally and socially (16), and has been considered an important component in patient care, interpersonal and communication skills, and, potentially, medical professionalism (17, 18). People with high emotional intelligence, it is hypothesized, are better able to read, understand, and react effectively to themselves and the emotional signals sent by others. These ‘soft’ skills, such as self-awareness, empathy, motivation, optimism, and effective interaction with others, define the ability of people to understand, reflect, and react to the variety of interpersonal situations of our complex social world.

The EI construct used in the work of Boyatzis and Goleman (19, 20) includes the competencies of self-awareness, self-management, social awareness, social relationships, and motivation; key elements in these domains directly relate to the ability to recognize and manage one's emotions in stressful situations (15, 16, 18, 21), understand and empathize with families/patients in high-stress settings, and communicate difficult issues effectively. In the business and academic worlds, EI correlates well with leadership success, job satisfaction, employee morale, job commitment, teamwork and financial success, and less work/family conflict (21). In the context of healthcare providers, high EI has been reported to positively contribute to better doctor–patient relationships, increased empathy, better teamwork, and effective stress management (22, 23), as well as increased clinical skill and overall physician performance (17, 24, 25).

However, there is little literature relating EI to successfully communicating bad news to patients. In this pilot project, we sought to explore the relationship of EI to pediatric interns’ bad news delivery skills in a simulated standardized patient (SP) setting, with the hypothesis that incoming residents with higher levels of EI would perform better at this complex skill.

## Methods

### Trainee population

All 44 PGY-1 pediatrics and medicine–pediatrics residents from a single, freestanding tertiary-care children's hospital were invited to participate in this study. The selection criteria were availability and willingness to participate. Although all 44 agreed to participate, scheduling conflicts during SP sessions precluded complete data collection for four residents; thus, our analyzable sample is based on 40 participants: 34 pediatric residents (28 female, 6 male) and 6 medicine–pediatrics residents (4 female, 2 male). Consent was obtained to access pre-existing EI data, and approval was granted by our Institutional Review Board prior to trainee recruitment and SP encounters.

### SP scenarios

The SP scenarios and skill assessment tool were selected from a previously published teaching intervention called ‘The GRIEV_ING Death Notification Protocol’ (26, 27), which includes both resident case scenarios and detailed SP instructions. The scenario used in our study was the unexpected death of a young boy hit by a car while bicycling. The skills assessment tool used was part of the protocol published by Hobgood and associates (26).

### Rationale for GRIEV_ING protocol

The GRIEV_ING protocol was selected for various reasons. First, it was the only freely available tool containing detailed instructions on the educational session (including didactics and small group discussion), specific SP encounters, and methods of assessment. Second, this protocol has been used in different populations of learners, including emergency medicine residents, fourth-year medical students, and emergency medical services providers (26–29). Last, it includes a pediatric death notification scenario used in our SP encounters, recognizing that the simulation would prove cognitively and emotionally challenging to study participants.

### SP encounters

A small local grant was used to compensate experienced SPs provided by The Clinical Skills Education and Assessment Center at the Ohio State University College of Medicine. The death notification was delivered by the resident to one of two SPs trained to play the role of grandparent. All encounters occurred in unused hospital patient rooms and were videotaped for later review. As per the protocol, after learning the details of the case, each resident had up to 15 min to deliver the news of the young boy's death to the surviving relative.

### SP data collection and analysis

SP encounters were conducted midway through the first year of residency training. Videotaped encounters were reviewed and scored independently by two investigators (SR, RS) over the ensuing 6-month period. The investigators had no prior experience in delivering bad news to the residents and had no knowledge of their EI assessments. Subjects’ final scores for each parameter were derived from the average of the two raters. As per the GRIEV_ING Protocol, each assessment item was scored on a yes/no basis, with residents receiving a ‘1’ for performing a particular skill and a ‘0’ for lack of performance of skill. Residents’ overall scores were determined based on the percentage of items performed correctly.

The intraclass correlation coefficient was used to assess inter-rater reliability.

### EI assessment

EI was assessed using an established measure, the Emotional and Social Competence Inventory (ESCI), which subjects completed during the final quarter of their PGY-1 year. The ESCI was developed by Richard Boyatzis and the Hay Group, in conjunction with Goleman (19, 20) (available at: www.haygroup.com), and involves the multisource assessment of the frequency of observation of 72 *behavioral* items – measured on a 5-point Likert scale ranging from ‘Never’ to ‘Always’, with identical assessment forms completed by the subject and 5–8 relevant peers/supervisors.

This tool has been used in the business literature (30) and further defined by Boyatzis (31), and is easily administered and applicable in defining the behaviors of individuals working in complex systems. The ESCI instrument was provided *pro bono* for research purposes by the Hay Group, who did not participate in this project in any way.

In the only reported comparable use of the ESCI, Webb used it to assess the EI and interpersonal/communication skills of a group of family medicine residents (18). In this study, residents’ achievement orientation and self-awareness were rated the highest and the lowest, respectively. Aside from coach/mentor and teamwork ratings, ESCI competencies among this group exceeded those from a North American sample of 1,557 workers (18).

We administered the ESCI self-assessment to residents online, with eight relevant peers/supervisors invited to complete a confidential assessment of the subject (resident). For each resident, a minimum of five other assessments were obtained and averaged to create the *Other* assessment. After reverse-scoring of designated items, scores were generated in 12 separate behavioral domains/competencies (Table 1). We used the averaged *Other* assessment rather than resident self-assessment in our analysis, because the assessment of EI by others was felt to be more relevant to communicating with others, specifically in bad news scenarios. Coefficient alpha was used to measure the internal consistency/reliability of each ESCI subscale.

**Table 1:** Comparisons between other and Self Emotional and Social Competence Inventory (ESCI) scales

ESCI scales (*N*=43)	Other	Self	Correlation	Dependent t-test	*P*

	Mean (SD)	Mean (SD)			
Emotional self-awareness	4.43 (0.20)	4.09 (0.60)	0.03	3.56	0.001
Achievement orientation	4.69 (0.13)	4.43 (0.43)	−0.05	3.85	0.001
Adaptability	4.48 (0.20)	4.07 (0.52)	−0.23	4.49	0.001
Emotional self-control	4.52 (0.22)	4.11 (0.46)	0.04	5.40	0.001
Positive outlook	4.53 (0.23)	4.20 (0.53)	0.06	3.69	0.001
Empathy	4.46 (0.20)	4.17 (0.50)	0.22	3.80	0.001
Organizational awareness	4.64 (0.18)	4.50 (0.51)	0.09	1.88	0.067
Conflict management	4.34 (0.24)	3.92 (0.63)	−0.00	4.10	0.001
Coach and mentor	4.38 (0.24)	3.93 (0.50)	−0.06	5.22	0.001
Influence	4.38 (0.20)	3.98 (0.58)	−0.03	4.25	0.001
Inspirational leader	4.42 (0.25)	3.81 (0.70)	0.03	5.36	0.001
Teamwork	4.67 (0.16)	4.49 (0.42)	−0.13	2.49	0.012

## Results

### GRIEV_ING protocol score

In assessing the consistency of GRIEV_ING Total scores, the inter-rater reliability of a randomly selected single rater was 0.66, while inter-rater reliability using the average of the two raters’ scores was 0.80, yielding substantial correlation based on Landis and Koch's characterization (32). In general, residents’ performance scores were low: Using the average of the two raters’ scores, the mean (standard deviation) of the 39 residents was 38.4 (9.0). The highest score was 55.2 while the lowest was 19.0. Internal consistency of the GRIEV_ING Total scale was adequate (*α*=0.66).

### EI scores

Residents’ *Other* EI mean scores were high (Table 1) relative to the ratings of previously published samples (18). No significant differences were noted in mean *Other* ESCI scores between 1) female and male residents or 2) pediatric and medicine–pediatrics residents. The internal consistency of each ESCI subscale for *Other* assessments was consistently high. Correlations between all subscales of the ESCI were significant (data not shown). Correlation was significant at < 0.01 (two-tailed) between all subscales, except for correlations between Coach/Mentor and Emotional Self-Control and Coach/Mentor and Positive Outlook, which were both significant at<0.05 (two-tailed). No ESCI subscales were significantly associated with residents’ death notification skills (GREIV_ING total score) (Table 2).

**Table 2:** Coefficient alpha for GRIEV_ING total and Emotional and Social Competence Inventory (ESCI) other scales and correlations between scales

	GRIEV_ING Total (*N=*39)	ESCI Other scales (*N*=43)											

		Emotional self-awareness	Achievement orientation	Adaptability	Emotional self-control	Positive outlook	Empathy	Organizational awareness	Conflict management	Coach and mentor	Influence	Inspirational leader	Teamwork
GRIEV_ING total	0.663												
Emotional self-awareness	0.010	0.863											
Achievement orientation	0.042	0.539^a^	0.791										
Adaptability	0.222	0.693^a^	0.658^a^	0.893									
Emotional self-control	0.086	0.437^a^	0.458^a^	0.799^a^	0.926								
Positive outlook	0.060	0.590^a^	0.434^a^	0.738^a^	0.770^a^	0.939							
Empathy	0.070	0.693^a^	0.586^a^	0.767^a^	0.673^a^	0.767^a^	0.871						
Organizational awareness	0.007	0.671^a^	0.463^a^	0.769^a^	0.462^a^	0.499^a^	0.673^a^	0.923					
Conflict management	0.185	0.757^a^	0.602^a^	0.815^a^	0.623^a^	0.683^a^	0.727^a^	0.659^a^	0.863				
Coach and mentor	0.128	0.580^a^	0.488^a^	0.589^a^	0.360^b^	0.366^b^	0.507^a^	0.599^a^	0.527^a^	0.900			
Influence	0.152	0.750^a^	0.593^a^	0.801^a^	0.456^a^	0.558^a^	0.649^a^	0.772^a^	0.808^a^	0.753^a^	0.845		
Inspirational leader	0.078	0.800^a^	0.593^a^	0.767^a^	0.458^a^	0.615^a^	0.645^a^	0.699^a^	0.784^a^	0.913^a^	0.831^a^	0.887	
Teamwork	0.014	0.641^a^	0.599^a^	0.814^a^	0.792^a^	0.815^a^	0.798^a^	0.644^a^	0.674^a^	0.662^a^	0.649^a^	0.690^a^	0.860

Diagonal values indicate coefficient alpha for each scale. Values below the diagonal indicate Pearson correlations between scales.

a Correlation is significant at the 0.01 level (two-tailed).

b Correlation is significant at the 0.05 level (two-tailed).

## Discussion

Our results demonstrate that first-year pediatric and medicine–pediatric residents have modest-to-low skills in death notification as assessed by SPs in a standardized setting. While not entirely surprising, these data do confirm the need to educate and further develop this complex skill. Based on the importance of this task (33–35), improving these skills may ultimately positively impact the doctor–patient relationship. Moreover, Common Program Requirements specified by the Accreditation Council for Graduate Medical Education (ACGME) state that each residency program must require its residents to achieve competency in these elements (36–39).

Contrary to our hypothesis, we found no correlation between residents’ abilities to deliver bad news and their other-assessed EI. While residents’ novice skills in this arena were predictable, we anticipated that those with higher EI would be comparatively better at this admittedly challenging task. That no aspect of EI was significantly correlated with GRIEV_ING Scores (but inter-correlated among one another) suggests that while the two measures apparently tap different constructs, they share little linear association.

Our guiding interest in this research question arose from recognizing the importance of communication skills to patient satisfaction and outcomes (33–35, 40–42). As prior research has shown modest relationships between EI and various communication skills (24, 25, 43), we speculate that the lack of a similar association here is due to the highly specific and complex nature of the task. Simply put, ‘successfully’ conveying bad news requires more than simply the practitioner's ability to recognize and regulate emotions.

In comparison, Cherry and colleagues (43) demonstrated that a specific dimension of EI, the ability to regulate emotions, predicted performance in a series of more basic communication skills among medical students. Since evidence exists that aspects of EI crucial to communication skills (e.g., empathy) can be developed (26, 27, 29, 44, 45), our study underscores the need to develop effective educational methods for teaching more complex communication skills. Coupled with previous findings suggesting that breaking bad news is an area with much room for improvement (1, 2, 4, 46, 47), our data should further motivate medical educators to pursue this element of resident education.

An additional point of discussion involves the challenge of balancing teaching, experiential learning, and deliberate practice in the context of duty hours and optimal patient care. Medical hierarchy and supervision, while necessary, can impact the experiences of physicians-in-training. For instance, our simulated scenario involving interns is unlikely to occur in a real-life pediatric emergency department; rather, the delivery of a patient's death is the responsibility of the attending physician. While this is considered the appropriate ‘standard of care’, trainees only become experienced by practicing this skill set. The use of SPs and simulate scenarios is one such mechanism for experientially learning medical communication skills (14, 35).

Limitations of this study include a small sample size drawn from a single institution, the use of a single SP encounter, the narrow range of performance scores in pediatric interns, and a reliance on only one type of bad news scenario (i.e., death notification) which is arguably among the most difficult. In addition, we could not account for differences in trainees’ previous experiences and/or training in breaking bad news and death notification. Last, our use of the ESCI does not necessarily ensure that similar results would be obtained with different EI measures.

While we did not find EI to correlate with demonstrated death notification skills, it is possible that EI is positively correlated with learners’ ability to improve and/or retain this skill. Exploring this possibility may provide insight into the utility of a more personalized approach to trainee education. In summary, results of this pilot study have further enhanced our appreciation of communication skills training as a critical component of residency education, leading to the development of a communication curriculum at our institution. While the delivery of bad news is a difficult and important element of doctor–patient communication, future plans are to include development in other challenging communication tasks, such as sensitive history-taking in adolescents and communication with angry families.
